# Monoamines, BDNF, Dehydroepiandrosterone, DHEA-Sulfate, and Childhood Depression—An Animal Model Study

**DOI:** 10.1155/2009/405107

**Published:** 2009-10-18

**Authors:** O. Malkesman, T. Asaf, L. Shbiro, A. Goldstein, R. Maayan, A. Weizman, N. Kinor, E. Okun, B. Sredni, G. Yadid, A. Weller

**Affiliations:** ^1^Interdisciplinary Program in the Brain Sciences, Bar-Ilan University, Ramat-Gan 52900, Israel; ^3^Department of Psychology, Bar-Ilan University, Ramat-Gan 52900, Israel; ^4^Biological Psychiatry Laboratory, Felsenstein Medical Research Center, Beilinson Campus, Sackler Faculty of Medicine, Tel Aviv University, Petah Tikva 49100, Israel; ^5^Faculty of Life Sciences, Bar-Ilan University, Ramat-Gan 52900, Israel; ^2^The Gonda (Goldschmied) Multidisciplinary Brain Research Center, Bar-Ilan University, Ramat-Gan 52900, Israel

## Abstract

Basal levels of monoamines and DHEA in four main limbic brain regions were measured in prepubertal Wistar Kyoto (WKY) rats (a putative animal model of childhood depression). Basal levels of “Brain-Derived Neurotrophic Factor (BDNF)” were also determined in two regions in the hippocampus, compared with Wistar strain controls. In the second phase, we examined the responsiveness of prepubertal WKY rats to different types of chronic antidepressant treatments: Fluoxetine, Desipramine, and dehydroepiandrosterone sulfate (DHEAS). WKY prepubertal rats exhibited different monoamine levels in the limbic system, reduced DHEA levels in the VTA and lower levels of BDNF in the hippocampus CA3 region compared to controls. In prepubertal WKY rats, only treatment with DHEAS produced a statistically significant decrease in immobility, compared to saline-administered controls in the forced swim test. Wistar controls were not affected by any antidepressant. The results imply that DHEA(S) and BDNF may be involved in the pathophysiology and pharmacotherapy of childhood depression.

## 1. Introduction

Major depression in children and adolescents is common, recurrent, and associated with significant morbidity and mortality [1]. The point prevalence of depressive disorders is 1%–2% of prepubertal children and 3%–8% of adolescents, with a lifetime prevalence of approximately 20% by the end of adolescence (for review see [2]).

Despite similarities in the clinical picture and longitudinal course of major depression in children, adolescents, and adults [3], there are notable differences in the neurobiological correlates and treatment response of depressed patients in these different age cohorts [4]. Depressed children and adolescents do not show evidence of hypercortisolemia as frequently as is reported in depressed adults [5], and most notably, depressed children fail to respond to antidepressant treatment as well as adults do [6]. For example, most of the trials with tricyclic antidepressants in both children [6] and adolescents [6, 7] have not been shown to be more efficacious than placebo, and only 3 (20%) of 15 antidepressants trials submitted to the FDA for pediatric depression demonstrated superiority of drug over placebo [8]. These differences between adult and childhood depression may indicate that some aspects of pathophysiology in monoaminergic circuits are unique to childhood depression [9]. 

Current antidepressant medications are based mostly on brain monoamine modulation. The weak response of depressed prepubertal children to conventional, clinically used antidepressants in contrast to adults [6] might imply that other non-monoaminergic factors may affect childhood depression. 

In the current study, we examined candidate novel mechanisms (with possible antidepressant potential) for childhood depression by using a putative genetic animal model for childhood depression: Wistar Kyoto (WKY) rats, derived from Wistar rats [10–12]. WKY prepubertal rats exhibited depression-like symptoms in a battery of behavioral tests and physiological measurements in earlier studies in our laboratory; including: anhedonic behavior [10]; long immobility duration in the swim test [11]; hypercortisolism [11]; and in infant rats-abnormal emotion-regulation capacities [12, 14]. Hence, the use of these young animals for studying potential mechanisms and antidepressant approaches appears to be appropriate and particularly relevant for exploring new treatment strategies for childhood depression. It is important to mention that though earlier findings from our laboratory suggest that though prepubertal FSL and WKY rats are *both* validated genetic animal models of childhood depression, they are fundamentally different. It seems that we have two different subgroups of animal models for childhood depression modeling two distinct clinical depressive syndromes (DMS-IV) that can also be found in depressed children and adolescents [15]. In general, the data on the WKY rats would seem most consistent with a melancholic depression profile. This is based on two characteristics: hypercortisolism—most consistent neuroendocrine alteration observed in patients with melancholic depression [16] and anhedonic-like behavior—the core symptom of the melancholic subtype of major depressive disorder [17]. On the other hand, data on the FSL rats would seem most consistent with atypical depression [18]—showing less classical symptoms of depression and showing no comorbidity of depression and anxiety [10, 11]. In the current study, we chose to focus on the more prototypical depressive profile—melancholic depression. Thus, the use of WKY prepubertal rats seems to be appropriate and particularly relevant in order to explore potential relevant brain mechanisms and to suggest potential new treatment strategies for childhood depression.

Though it is believed that depletion of serotonin and/or dopamine is one of the causes of depression [19], and depression can be treated by drugs increasing this activity [20], the monoaminergic hypothesis has not been able to provide adequate explanations for the pathogenesis of depression [21]. 

In recent years, it has been shown that Dehydroepiandrosterone (DHEA) and its sulfate ester (DHEAS) play a role in the neurobiology of depression (for review, see [22]) and even possess antidepressant-like properties [23, 24] that can be explained by the interaction between the sigma 1 receptor agonist DHEA(S) [25] and noradrenaline and serotonin neurotransmission. A recent review suggests that neurosteroids, including DHEA and DHEAS, may be involved in the pathophysiology and pharmacotherapy of a variety of disorders in children and adolescents, including depression [26]. Accordingly, concentrations of DHEA and DHEAS in humans typically decrease steadily with age [22] and it has been proposed that DHEA as well as DHEAS may play a role in neurodevelopment, due to a transient expression of steroidogenic enzyme (P450c17) and the potential ability of DHEA(S) to regulate neuronal pathway formation [22, 27]. Thus, DHEA(S) may potentially play an important role in (childhood and adult) depression, both because of its antidepressive effect and its role in neuronal pathway formation.

The neurotrophic factor hypothesis is one of the latest theories of the pathophysiology of depression. Neurotrophic factors were first characterized for regulating neural growth and differentiation during development, but are now known to be also potent regulators of plasticity and survival of adult neurons and glia [28]. The “neurotrophin hypothesis of depression” is based largely on observations that decrease in hippocampal BDNF levels are correlated with stress-induced depressive behaviors and that antidepressant treatment enhances the expression of BDNF (see [29] for review). Therefore, the BDNF hypothesis postulates that a loss of BDNF is directly involved in the pathopysiology of depression, and that its restoration may underline the therapeutic efficacy of antidepressant treatment [30].

Several lines of evidence suggest that BDNF might be an important agent of therapeutic recovery from depression, and it might also provide protection against stress-induced neuronal damage [28, 31]. Chronic (but not acute) administration of virtually all classes of antidepressants increased BDNF expression in the dentate gyrus and the pyramidal cell layer of the hippocampus in rodents [32]. The findings could also explain why antidepressant response is delayed: it would require sufficient time for levels of BDNF to gradually rise and exert their neurotrophic effects [28]. Furthermore, Shirayama et al. [33] demonstrated that a single bilateral infusion of BDNF into the dentate gyrus of the hippocampus produced an antidepressant effect in two (behavioral) animal models of depression: learned helplessness and the forced swim test. In addition, though BDNF and serotonin are two seemingly distinct signaling systems that play regulatory roles in many neuronal functions, a common feature of the two systems is their ability to regulate the development and plasticity of neuronal circuits involved in mood disorders such as depression and anxiety. BDNF promotes the survival and differentiation of serotonin neurons. Conversely, administration of SSRIs enhances BDNF gene expression. There is also evidence for synergism between the two systems in affective behaviors and genetic epistasis between BDNF and the serotonin transporter gene (see [34] for review).

In animal models, it has been shown that stress (and high levels of glucocorticoids) may give rise to atrophy of the hippocampal neurons in the CA3 subregion [35]. In addition, it has been shown that stress paradigms decrease adult neurogenesis in the hippocampal dentate gyrus [36].

In sum, the hypothesis of BDNF and depression suggests that if BDNF is no longer made in appropriate amounts, instead of the neuron prospering and developing more and more synapses, stress causes vulnerable neurons in the hippocampus to atrophy and possibly undergo apoptosis when their neurotrophic input is cut off. This, in turn can lead to depression and to the consequences of repeated depressive episodes [19].

Accordingly, due to the findings that depressed children respond less well to antidepressant treatments, and based on the connections between serotonin, DHEA and BDNF and their role in neurodevelopment, we examined their levels in an animal model of childhood depression. 

In the main study, in order to identify unique brain areas that might be implicated in children suffering from depression, we measured basal levels of monoamines and DHEA in subcortical structures of the limbic system, which have been found to play critical roles in depression [37]: the nucleus accumbens (NAc), the hypothalamus, the ventral tegmental area (VTA), and the amygdala. These areas have been found to play a critical role in domains which are prominently affected in most depressed patients such as regulation of motivation, sleep, and responses to pleasurable and aversive stimuli [28]. In addition, we measured basal levels of BDNF in the CA3 and the dentate gyrus (DG) in prepubertal WKY rats and their Wistar controls.

In a second study, we examined how the WKY strain responds to different antidepressant treatments. We administered two different clinically used antidepressants that influence the brain monoaminergic system: fluoxetine (a selective serotonin reuptake inhibitor; SSRI) and desipramine (nonselective norepinephrine reuptake inhibitor) and explored their effect on prepubertal rats from the WKY strain and their Wistar controls. In addition, we employed DHEAS as a potential antidepressant treatment (while being aware that it is a steroid that could potentially influence the pattern of sexual maturation). The effectiveness of all three different treatments was assessed by a behavioral test often used for screening antidepressants: a modified version of the forced swim test of Porsolt et al. [38]. The animals were studied at the age of 34-35 days, because at this developmental phase, before sexual maturity, rats are able to show immobility in the forced swim test [39]. This age also allowed for a 2-week treatment period, starting from the day of weaning.

## 2. Methods

### 2.1. Animals

Adult WKY and Wistar rats were bred in our colony at Bar-Ilan University's Specific Pathogen Free colony at the Gonda Brain Research Center. After weaning the pups were housed in a polypropylene cage (18.5 cm height × 26.5 cm width × 43 cm length), three per cage, in a temperature controlled vivarium (20–22°C), under a 12h-12h light : dark cycle (lights on at 0700). Food and water were available *ad libitum*. Several studies showed that repeated short-lasting stress induced by handling alters the action of the HPA axis [47, 48] and that mild stress of daily injections alone may alter morphology of different brain regions [49]. Therefore, in the current study, osmotic mini-pups (loaded with the antidepressant drugs or vehicle) were implanted only in the male rats (PND 21) that were intended to participate in the antidepressants experiment. 

The animals were studied, before sexual maturity, at the age range of 30–35 days. At this developmental phase, rats are able to show immobility in the swim test [39], and their developing HPA system is responsive, as the “stress hyporesponsive period” is behind them [50]. This developmental profile is roughly similar to the prepubertal human developmental profile [51].

The study protocol was approved by the Institutional Animal Care and Use Committee, is in accordance with the National Institutes of Health guide for the care and use of Laboratory animals (NIH Publications no. 8023, revised 1978) and adhered to the guidelines of the Society for Neuroscience.

### 2.2. Brain Dissection and Extraction

At the age of 34-35 days, 12 male rats (4–6 of each line) were decapitated and their brains removed rapidly, and dissected as previously described [52]. Since DHEA is a stress-responsive hormone [22], extra steps were made in an attempt to prevent pre-decapitation stress: rats were waiting outside of the decapitation room, after each animal's decapitation gloves were changed, and all the equipment was cleaned. Briefly, the entire hypothalamus was surgically dissected out with forceps and frozen immediately at −80°C. The brains were then placed in a rat brain mold (constructed at Bar-Ilan University) on ice, and serial 0.5 mm sections were cut and placed on chilled microscope slides. Tissue punches (NAc, hypothalamus, VTA, Amygdala, DG, and CA3) were taken rapidly, using a stainless steel cannula with an inner diameter of 0.6 mm. The tissue samples were frozen immediately at −80°C. Extraction was achieved by thawing the punches and subjecting them to probe sonication (80 W for 5 s with a B-12 Sonifier; Branson, Danbury, Conn, USA) in 0.3 mL of PBS on ice. A sample (10 *μ*L) was removed for protein analysis and the rest was subjected to centrifugation (2,000 g, 10 min, 4°C). The resulting supernatants (the tissue extracts) were divided into two separate tubes: 90 *μ*L for DHEA determination and 60 *μ*L for monoamine determination. To the tube containing the supernatants for the monoamine determination we added 120 *μ*L PCA and then filtered it (0.45 *μ*m Acrodisk; Gelman, Ann Arbor, Mich, USA). Both of the tubes were stored at −80°C until used for the determination of monoamines (HPLC) or DHEA (RIA).

### 2.3. Analysis of Monoamine Content in the Tissue Punches

Quantitation of the 5-hydroxytryptamine (Serotonin/5-HT), 5-hydroxyindoeactic acid (5-HIAA), homovanillic acid (HVA), dopamine (DA), 3,4-dihydroxy-phenylalanine (L-DOPA), and dihydroxyphenylacetic acid (DOPAC) content of the tissue punch extracts was performed as described previously [52]. Briefly, the filtered supernatants of each tissue extract were injected directly via an HPLC pump (Model 515, Waters, Milford, Mass, USA) onto a column (Merck Chemicals, Ltd; c-18, 5 *μ*m particle size, 4.6 mm id X 250 mm, 30°C) coupled to an electrochemical detector (Digital Electrochemical Amperometric Detector, Antec-Leyden, Zeoterwoude, the Netherlands), and the oxidation potential was set to 0.76 V. The mobile phase (0.55 g heptane sulphonic acid, 0.2 g EDTA, 16 mL triethylamine, 12 mL 85% phosphoric acid, and 40 mL acetonitrile in 2 L of water; pH 2.6) was pumped at 0.8 mL/min. Monoamine and metabolite concentrations were expressed in relation to the protein content of the samples, which were quantified with Bio-Rad Protein Assay Kit. Results are presented in pmol/mg protein.

### 2.4. DHEA Determination

DHEA level was measured using a DSL 9000 Active DHEA-coated tube radioimmunoassay (RIA) kit (Diagnostic Systems Laboratories, Webster, Tex, USA). 0.75 mL limbic region homogenates were extracted twice with 1 mL diethylether, centrifuged at 350 g for 5 minutes and kept for about 15 minutes at −70°C to allow the aqueous phase to freeze. The etheric phase was decanted into a new glass tube, evaporated till dryness and dissolved in 120 *μ*L of standard 0 of the RIA kit. 100 *μ*L were used for the determination of DHEA [53]. The detection limit of the assay is 0.07 nmol/L; assay variability is 10.2% between runs and 5.6–10.6% within runs according to the level of DHEA in the sample; cross-reactivity with other steroids is <0.2%. Results are presented in pmol/mg protein.

### 2.5. BDNF Determination

BDNF levels were determined using BDNF Emax ImmunoAssay System (Promega Corporation, Madison, Wis, USA) as previously described [54].

### 2.6. Antidepressants Administration

At PND 21–23, anti-depressant treatments were administered. Osmotic mini-pumps (Alzet, Model 1002, 0.25 *μ*l per hour, 14 days) were filled with the drugs (Fluoxetine, Desipramine, DHEAS—all in concentrations of 8 mg/kg initial BW/day) or saline, and implanted subcutaneously in the rats from the 2 strains, under anesthesia (Nembutal 30 mg/kg). We preferred the sulfate formation (DHEAS), because it dissolves with water, which allows the use of osmotic pumps, as opposed to DHEA that dissolves with oil. 

 The dose of the antidepressant treatments was chosen according to several studies conducted on adult rats from this strain [55–57]. Different doses were used in these studies ranging from 5–10 mg/kg. We chose the dose of 8 mg/kg since this dose is in the middle of the range found effective in these studies and due to the fact that this study deals with prepubertal rats that might show over-sensitivity to the higher doses of the antidepressant treatments. After two weeks in which the pumps slowly released the drugs and the rats' body weight increased dramatically, individual doses ranged from 3.2–4.94 mg/kg, with no significant between-group differences in dose. One way ANOVA on the different treatments at the different ages (PND = 21 and 35) of the two strains (Wistar versus WKY) further showed no significant differences between the strains in pattern of decrease in dose over time [F(7,35) = 0.69; NS]. At the age of 34-35 days, the forced swim test procedure was conducted (*N* in each group = 8).

### 2.7. Swim Test

The forced swim test developed by Porsolt et al. [38] has become a widely used paradigm for studying stress responses and screening antidepressant drugs ([39]; but see [40]). Prolonged immobility duration in this test is sometimes regarded as behavioral despair, an animal analogue of human depression. The general procedure in this paradigm is to immerse rats or mice in a cylinder of water from which there is no escape. Twenty-four hours later, rats are retested for 5 minutes. Typically, animals paddle vigorously when first immersed, then become relatively immobile and adopt a characteristic vertical floating response. When observed during the retest period, they are more immobile than during their initial immersion. In some variations of the test, rats are only immersed once and immobility time is recorded during this one-time only session [41, 42]. Abel [39] has demonstrated that the vertical immobility response in the forced swim test has a sudden onset, beginning at 21 days of age and quickly stabilizing at 26 days of age. Overstreet [43] and Yadid [44] have shown longer immobility durations in adult Flinders Sensitive Line (FSL) male rats compared to controls, using the modified “Porsolt” paradigm—a one-session procedure. In addition, the predictive validity of this modified paradigm has been supported is several studies (e.g., [45, 46]). Using the same procedure, in a reduced-size apparatus designed for prepubertal rats [39], we similarly described longer immobility time in prepubertal FSL and WKY rats, compared to their control strains [11].

At the age of 34-35 days, after two weeks of anti-depressant treatment, each male rat was weighed and then immersed in a Plexiglas cylinder designed especially for the size of prepubertal rats (height = 45.5 cm, diameter = 14.0 cm) filled to 24 cm with fresh tap water heated to 34 ± 1°C, for 5 minutes [39]. No animal was tested more than once. Duration of floating/immobility behavior was measured. The criterion for floating: making only the minimal movements necessary to keep the head above water, with no forelimb movements. The water was changed between test animals. All tests were performed in a dark room and during testing the Plexiglas cylinder was illuminated by two 25-W red light bulbs, placed approximately 50 cm above the cylinder. All tests were conducted between 10:00–13:00 hour.

### 2.8. Data Analysis

Quantification of the 5-HT, 5-HIAA content of the tissue punches extracts from each brain region (Amygdala, VTA, NAc, and hypothalamus) was analyzed by independent *t*-tests comparing the WKY and Wistar strains (with a Bonferonni correction).

Quantification of the HVA, DA, DOPA, DOPAC content of the tissue punches extracts from each brain region (Amygdala, VTA, NAc, and hypothalamus) were analyzed by independent *t*-tests comparing the WKY and Wistar strains (after a Bonferonni correction).

DHEA basal levels were analyzed by independent *t*-tests comparing the WKY and Wistar strains, separately for each brain region (Amygdala, VTA, NAc, and hypothalamus).

BDNF basal levels were analyzed by independent  *t*-tests comparing the WKY prepubertal rats and their Wistar controls, separately for each brain region (CA3 & DG).

Immobility in the forced swim test and weight at PND 21 and 35 (the latter: the day in which the forced swim test took place) of animals from the two strains (WKY and Wistar) that received chronic administration of saline were compared by *t*-tests for independent samples. This was in order to compare the results from the current study with former results from our lab, in which strain differences were found [11]. 

In the second phase, antidepressant treatment differences were analyzed by two-way (strain X treatment) univariate analyses of variance (ANOVA), followed by one way ANOVAs, performed separately in each of the two strains, with treatment (desipramine, fluoxetine, DHEAS versus saline) as the independent variable. In these ANOVAs, immobility duration in the swim test, weight at PND 21 and weight at PND 35 were the dependent variables. The ANOVAs were followed by Tukey's HSD post-hoc tests, examining the effects of the different antidepressant treatments.

## 3. Results

The basal levels of monoamines in the different brain regions are presented in Table 1. As evident from Table 1, WKY prepubertal rats exhibited significantly lower levels of 5HIAA in the Nac (*t*(9) = 3.181; *P* < .05) compared to their control line. A non-significant tendency was apparent also in HVA levels (*t*(8) = 1.925; *P* < .1). In the VTA, the WKY rats exhibited higher levels of DOPA (*t*(5) = 4.84; *P* < .01) compared to their controls. WKY rats exhibited significantly higher levels of DOPAC (*t*(8) = 6.97; *P* < .01) in the hypothalamus, compared to their Wistar controls. Two other apparent differences in this brain region, lower levels of 5HIAA (*t*(7) = 2.399, *P* < .05) and a tendency towards higher levels of DOPA (*t*(7) = 2.139; *P* < .1) in WKY compared to Wistar rats, did not reach statistical significance, given the Bonferonni correction. In the Amygdala, WKY rats showed a nonsignificant tendency toward higher levels of 5HIAA (*t*(8) = 2.355; *P* < .05, Bonferonni correction = NS) compared to Wistar controls.

**Figure 1:**
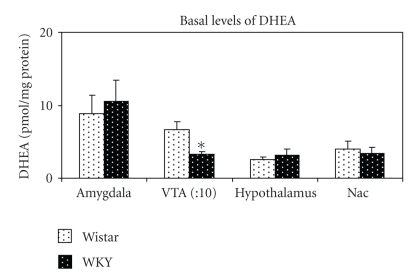
Mean basal levels of DHEA (± SEM) of 34-35 day old Wistar and WKY rats. Levels in the VTA are divided by ten for presentation purposes. *P < .05.

The basal levels of DHEA in the different brain regions are presented in Figure 1. WKY rats exhibited significantly lower levels of DHEA in the VTA compared to their control line-Wistar (*t*(5) = −2.605; *P* < .05). No significant differences in DHEA levels were found in the other brain regions.

**Figure 2:**
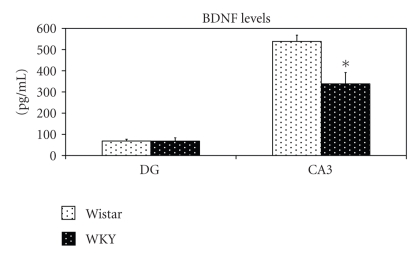
Mean basal levels of BDNF in the DG and CA3 (± SEM) of 34-35 day old Wistar versus WKY rats (*N* = 5-6 each group). *P < .05.

WKY prepubertal rats exhibited lower levels of BDNF in the CA3 (*t*(7) = 2.73; *P* < .05) compared to their Wistar controls, while no significant differences were found in the DG (*t*(4) = 0.02; *P* = ns); (Figure 2).

As evident from Table 2, *t*-tests for independent samples revealed that WKY rats weighed significantly less (*t*(14) = 2.83; *P* < .05) compared to Wistar controls (as was found in a former study in our lab; [11]). T-tests for independent samples also revealed that WKY rats at PND 35 exhibited longer immobility duration in the forced swim test (*t*(14) = 3.34; *P* < .05) compared to their Wistar controlled (Wistar mean immobility duration (sec): 115.625 ± 3.466; WKY mean immobility duration (sec): 244.37 ± 17.28; *P* < .05).

**Figure 3:**
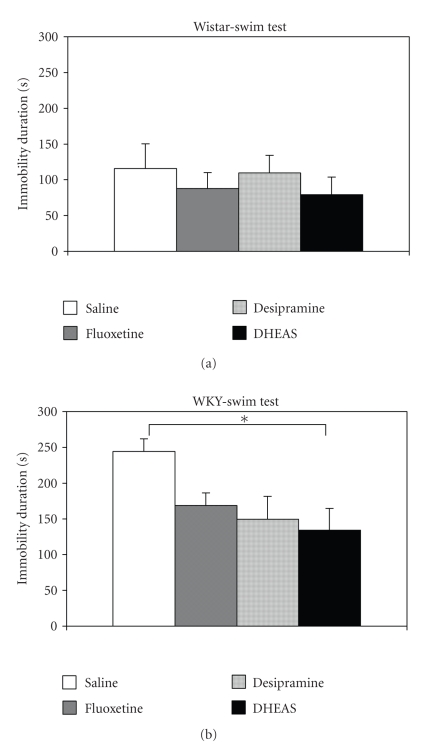
(a) Mean immobility duration in sec. (± SEM) of 35 days old Wistar rats after chronic administration of antidepressants (desipramine, fluoxetine, DHEAS) or saline (*N* = 8 in each group). (b) Mean immobility duration in sec. (± SEM) of 35 days old WKY rats after chronic administration of antidepressants (desipramine, fluoxetine, DHEAS) or saline (*N* = 8 in each group). *P < .05.

Analyzing the effects of the antidepressant treatment on swim-test immobility in the two strains by 2-way ANOVA showed that WKY rats were significantly more immobile (mean = 174 sec) than Wistar rats (mean = 98), F(1,56) = 17.06; *P* < .001; and that treatment with DHEAS, overall, significantly reduced immobility duration compared to saline treatment, F(3,56) = 2.84; *P* < .05. Tukey's post hoc test (*P* < .05) did not further show significant differences between the group that received saline and the groups that received desipramine or fluoxetine. The strain X treatment interaction was not statistically significant. One way ANOVA on immobility levels of the Wistar rats in the swim test revealed no significant effect for the treatment, F(3,28) = 0.41; *P* = N.S (Figure 3(a)). On the other hand, in the WKY rats, treatment with DHEA significantly reduced immobility duration compared to saline treatment, F(2,28) = 3.76; *P* < .05, Tukey's post hoc test did not further show significant differences between the WKY rats that received saline and those that received desipramine or fluoxetine (Figure 3(b)). 

Two-way ANOVAs on the rats' body weights on PND21 and 35 showed that Wistar rats weighed significantly more than WKY rats, as expected (PND21 : F(1,35) = 24.17, *P* < .001; PND35 : F(1,35) = 59.80, *P* < .001). However, there were no significant treatment differences or strain X treatment interactions. In both the Wistar and the WKY rats, at both ages, no significant differences in body weight were revealed between the treatment groups (PND21 : Wistar : F(3,15) = 0.33; *P* = NS; WKY : F(3,12) = 1.5; *P* = N.S; PND35 : Wistar : F(3,28) = 2.54; *P* = N.S; WKY: F(3,28) = 2.21; *P* = NS) (Table 2).

## 4. Discussion

In attempt to better understand the neurobiological basis of childhood depression, we explored, in the main study, monoamine and DHEA levels in the limbic system, and BDNF levels in the hippocampus. Next, upon the results, we performed a preliminary evaluation of a new and unique potential treatment approach for depressed children, namely, DHEAS, using prepubertal rats from a *putative* animal model of depression—the WKY strain and the Wistar strain as their control (—as suggested by the results from several studies conducted in our lab [10–12]). Finding effective antidepressant treatments in young WKY rats would also provide support for their predictive validity as an animal model for childhood/adolescent depression. 

Because of their unique importance and influence on depression, we focused on four subcortical areas in the current research: NAc, VTA, amygdala, and hypothalamus. Another important brain region which influences depression and should be mentioned is the hippocampus; (for reviews see [58, 59]). Following earlier findings [30] both in animal and human studies, and the connections between BDNF and serotonin in depression [34], we measured basal levels of BDNF in two major brain regions in the hippocampus: DG and CA3.

The results in the current study showed that WKY prepubertal rats had lower basal levels of DHEA, compared to their controls, in the VTA, a brain region suggested to be central in reward and motivation [37]. Though not much is known about the activity of the neurosteroid DHEA and DHEAS in the brain, increasing data indicate an antidepressant role for DHEA(S) (see [22] for review). In addition, a positive relationship between DHEA and monoamines was demonstrated; for example, DHEA administration caused a significant increase in serotonin levels in the PVN of the hypothalamus [60]. The antidepressive effect of DHEA may be triggered by interaction with several different receptors: GABA_A_ [61]; NMDA [62]; sigma-1 receptors [25, 63]. The sigma-1 receptors were demonstrated to elicit noradrenaline- and serotonin-neurotransmission and considered to have a role in the antidepressants effect [64, 65]. Lower levels of DHEA in regions of the WKY limbic system may explain some of the depression-like symptoms detected in these prepubertal rats, such as increased immobility time in the forced swim test and abnormal social play [11].

The results from the monoamine measurements showed that WKY prepubertal rats had lower levels of 5HIAA, compared to their controls, in the Nac. Both animal [66] and human (postmortem) studies [67] have revealed a close correlation between brain and cerebrospinal fluid (CSF) 5HIAA levels. Lowered CSF 5HIAA in depression was shown to correlate positively with increased anxiety [68] as also observed in the prepubertal WKY rats [10]. In addition, WKY rats showed higher levels of DOPA in the VTA and higher levels of dopamine metabolite (DOPAC) in the hypothalamus in WKY compared to Wistar prepubertal rats. 

Taking together the findings of low levels of DHEA in VTA of prepubertal WKY, together with the decreased metabolism of serotonergic neurons in the hypothalamus and Nac, as reported in other studies [60, 69] may indicate a connection between these brain regions and neurochemical mechanisms that contribute to “depression-like” symptoms found in this strain. This connection may involve the agonistic effects of DHEA(S) on the activity of sigma 1 receptors [22, 26, 60, 69], as well as on the neural network of these brain regions [70]. Future studies are needed to determine the effects of role neurochemical limbic network in the “depressive-like” behaviors exhibited by this strain.

Alternatively, functional connection between the serotonergic and dopaminergic system in the limbic circuit was suggested to play a role in depressive behavior [44]. Alterations in DHEA and limbic dopamine turnover were demonstrated recently [33], and serotonin receptors have been found activating a large population of dopaminergic neurons in the VTA [71]. Hence, DHEA may change serotonergic tone, which may lead to alteration in dopaminergic functioning as implied in depression [72]. The higher levels of DOPAC in the hypothalamus exhibited by the WKY prepubertal rats in this study may thus be explained by the abnormal levels of serotonin in these structures, as a result of abnormal DHEA levels in the VTA of theses animals.

From the BDNF results it seems that prepubertal WKY rats exhibit lower levels of BDNF in the hippocampus (at least in one region-CA3) as has been found in several human studies [73] and animal studies [30], and in accordance with the BDNF theory of depression.

Recently, a study measuring BDNF and TrkB levels in the hippocampus of prepubertal rats following antidepressants treatment showed increased BDNF protein and mRNA, as well as TrkB mRNA, at different ages (PND 13, 21, 28) [74]. The results from this study, together with the results from our study using an animal model of childhood depression [11], might indicate that BDNF has a key role in childhood depression.

However, it is noteworthy to mention, that the evidence for the involvement of BDNF in the pathophysiology of depression is currently inconsistent. On one hand, as described above, decreased BDNF levels are associated with both human depression and “depressive-like behavior” in rodent models of the disorder. A number of clinically effective antidepressants increase BDNF levels, while direct BDNF infusions and genetic overexpression demonstrate antidepressant-like activity. On the other hand, a number of pharmacological studies have generated negative results, while others describe findings directly contradicting a simple casual relationship between total brain BDNF levels and mood (see [30] for review).

In the second phase of the current study, we chronically administrated two different antidepressant treatments (fluoxetine and desipramine), DHEAS and saline to prepubertal rats from the two strains. Though *prepubertal* WKY rats treated by desipramine or fluoxetine appeared to exhibit lower immobility duration in the forced swim test, only treatment with DHEAS produced a significant decrease in immobility, compared to saline-administered controls. Wistar controls were not affected by any of the antidepressants. These results are in accordance with the lower levels of DHEA found in the VTA of the WKY rats in first phase of our study (This study, though, should be regarded as a preliminary examination, as a dose-response, multimeasure experiment with a greater number of subjects is clearly needed to clarify the relative potency of the different treatments). However, though DHEAS may be synthesized in the brain from DHEA, they are not the same hormones and they may act through different mechanisms [22]. Therefore one of the limitations of the current study is the use of DHEAS (and not DHEA) as a neurosteroid antidepressant treatment (due to the limitations of the osmotic minipumps) in the second phase of the study. 

Chronic administration of tricyclics, such as desipramine, improves most of the “depressive-like” symptoms exhibited by WKY adult rats [55, 75]. Other studies showed that selective 5HT reuptake inhibitors such as fluoxetine, only affect a portion of the serotonin-receptor binding capacity in the brain [56, 57]. The differences between the results in the current study and findings from other studies [75] might explain the weak response of depressed prepubertal children to conventional, clinically used antidepressants, in contrast to adults [6], emphasizing the pharmacological differences between adult depression and childhood depression, and the need for specific and novel therapeutic strategies for childhood depression. 

In addition, the current results indicate that young WKY rats receiving 14 d saline administration exhibited longer immobility duration in the forced swim test and weighed significantly less compared to Wistar controls, as in untreated naïve prepubertal rats [11]. We did not find treatment X group effects on body weight, at the onset (PND 21) and end (PND 35) of the study, in both Wistar and WKY rats. Although the dose selected was adjusted to the weight of the rat at the beginning of the study, this dose gradually changed during the experiment since the subjects' weights changed. However, analyzing the dose at the end of the study and the rate of change between the treated groups showed no significant between-group differences. Importantly, even though there was a gradual decrease in the dose over ontogeny, a significant effect on swim-test immobility was found.

High ratio of corticosterone/DHEA may be associated with depression (e.g., [76]). In previous studies in our laboratory, thirty-five-day-old WKY rats have displayed high levels of plasma corticosterone (CORT) and adrenocorticotropic hormone (ACTH) [11]. Hence, chronic administration of DHEAS in our study might have decreased the ratio of CORT/DHEA, consequently alleviating depression-like symptoms exhibited by the WKY prepubertal rats. However, one should consider that a disassociation might exist between DHEAS and ACTH, for example, during (chronic) stress or medical illness [77], and the fact that DHEA levels can be regulated independently of cortisol/corticosterone [78]. Thus, the DHEAS effect seen in this study may be explained by other neuromechanisms, such as enhancement of norepinephrine [79] and serotonin [80].

A recent study showed that DHEA can render an otherwise ineffective dose of fluoxetine capable to increase progenitor cell proliferation to the same extent as doses four times higher [81]. However, this synergistic action did not appear to be mediated by alterations in BDNF gene expression, or by TrkB, mineralcorticoid, glucocorticoid,o or 5HT1A receptor expression in the DG, or by altered levels of plasma corticosterone. We note that this study was conducted in brain samples, no behavioral paradigms were employed, and the samples were from wild-type rats—not “animal models of depression.” 

Former results suggesting an antidepressant-like effect of DHEA in adult rats of a different strain exhibiting “depression-like” symptoms [53] and humans [23] together with our current data suggest that the neurosteroids DHEA(S) may be a promising adjunct treatment approach for depressed adults and especially for depressed children and adolescents (while closely monitoring sexual maturation and HPA axis activity) who fail to respond to the available monoaminergic antidepressant treatments. According to our results showing abnormal basal levels of BDNF, together with the findings showing the interactions between serotonin, BDNF, and DHEA [34], it seems that BDNF might be a possible mediator of the DHEA antidepressive activity (the results of [81] notwithstanding). However, further studies need to be conducted in the future to determine this potential pathway. 

Please note that this paper does not attempt to suggest wide use of DHEA nor DHEAS as antidepressants for childhood depression but it only explores the possibility that DHEA(S), at low doses and/or in conjunction with clinically used antidepressants may have therapeutic potential in some cases. The use of this neurosteroid in depressed children and adolescents should be further evaluated and closely monitored in order to prevent unwarranted side effects. In addition, it is noteworthy to mention four major limitations of the current study: (1) there are differences between rodents and humans in brain DHEA expression—in rodents brain DHEA is derived mainly if not solely from local synthesis and not from peripheral synthesis, while in human beings, brain DHEA may be derived from both local synthesis and peripheral synthesis [22]. (2) DHEA and DHEAS concentrations typically decrease steadily with age [22]. Though earlier studies showed developmental differences between WKY and Wistar controls [12] indicating abnormal differences related to childhood depression, in the current study there were no direct comparisons between the prepubertal rats to older age groups, making it difficult to establish whether the observed effects are related to childhood depression per se or represent general differences between the WKY strain and their corresponding controls. (3) There are differences between rats and humans in the developmental course of DHEA and DHEAS—prepubertally, humans undergo adrenarche, the maturation of the adrenal gland, and after that the entire system is much different than in a truly juvenile state. Rats do not go through adrenarche, so the applicability of the rat as an animal model of DHEA-depression-development is questionable. (4) In an attempt to avoid the influence of gender and female sex hormones on developmental characteristics, only males were examined in the current study, therefore further studies need to be conducted in order to explore the involvement of DHEA(S) in childhood depression in females.

## Figures and Tables

**Table 1:** Mean basal levels of monoamines and their metabolites in the four different limbic brain regions (± SEM) of 34-35 day old Wistar and WKY rats (pmol/mg protein).

Brain Region	Strain	DOPA	DA	DOPAC	HVA	5HT	5HIAA
Nac	Wistar	7.03 ± 3.22	70.7 ± 7	55.6 ± 8.2	30.9 ± 1.4	2.95 ± 0.2	11.1 ± 1.28
	WKY	3.31 ± 0.95	69.6 ± 4	53.6 ± 2.6	26 ± 1.5^#^	1.9 ± 0.71	5.67 ± 1.05*

VTA	Wistar	2 ± 1.7	10.9 ± 5.1	16.57 ± 9.17	1.49 ± 0.68	3.48 ± 2.32	4.58 ± 2.96
	WKY	14.15 ± 1.7**	10.6 ± 6.7	3.12 ± 1.3	5.55 ± 4.8	2.48 ± 1.2	1.16 ± 0.83

Hypothalamus	Wistar	49.7 ± 6.14	197 ± 52.1	57.6 ± 2.9	30.4 ± 8.2	16.56 ± 4.4	114 ± 10.2
	WKY	88.2 ± 17.2^#^	96 ± 30.4	107.7 ± 7.7**	31.8 ± 9.5	17.08 ± 4.6	83.8 ± 5.7*

Amydgala	Wistar	11.84 ± 1.67	6.88 ± 1.2	2.59 ± 1	4.70 ± 1.1	2.25 ± 0.2	1.38 ± 0.8
	WKY	8.35 ± 1.2	7.91 ± 1.5	4.74 ± 1.2	2.86 ± 1.6	2.02 ± 1.1	4.29 ± 0.9*

**P < .01 Wistar versus WKY; *P < .05 Wistar versus WKY; ^#^ *P* < .1 Wistar versus WKY.

**Table 2:** Weight in grams (mean ± SEM) of 21-day-old and 35-day-old Wistar and WKY rats in the different treatment groups (DHEAS, desipramine, fluoxetine, saline).

Line	Age	Treatment	Weight (gr.)
Wistar	PND 21	Saline	58.6 ± 11.6
		Fluoxetine	56.6 ± 3.5
		Desipramine	56.5 ± 4.9
		DHEAS	55.5 ± 4.0
		Saline1	106.2 ± 6.6
	PND 35	Fluoxetine	123.9 ± 1.7
		Desipramine	115.7 ± 6.9
		DHEAS	123.1 ± 3.3
		Saline	38.8 ± 2.9

WKY	PND 21	Fluoxetine	33.6 ± 2.6
		Desipramine	29.6 ± 1.2
		DHEAS	34 ± 3.8
		Saline*¹*	82.1 ± 5.3
	PND 35	Fluoxetine	72.5 ± 4.7
		Desipramine	71.0 ± 3.0
		DHEAS	67.9 ± 2.7

^1^ *P* < .05 Wistar (saline) versus WKY (saline) at PND 35.
